# Validation of the Japanese Version of the Burnout Assessment Tool

**DOI:** 10.3389/fpsyg.2020.01819

**Published:** 2020-08-11

**Authors:** Keiko Sakakibara, Akihito Shimazu, Hiroyuki Toyama, Wilmar B. Schaufeli

**Affiliations:** ^1^Faculty of Sociology, Toyo University, Tokyo, Japan; ^2^Faculty of Policy Management, Keio University, Kanagawa, Japan; ^3^Faculty of Educational Sciences, University of Helsinki, Helsinki, Finland; ^4^Department of Social, Health and Organizational Psychology, Utrecht University, Utrecht, Netherlands; ^5^Department of Work, Organizational and Personnel Psychology, KU Leuven, Leuven, Belgium

**Keywords:** Burnout, assessment tool, job demands–resources model, validation, Japanese

## Abstract

The current study aimed to validate the Japanese version of the Burnout Assessment Tool (BAT-J), a new burnout measure. We conducted an Internet survey to confirm the validity and reliability of the BAT-J, using registered monitors from a Japanese survey company. The first-wave survey was conducted in May 2018, with 1,032 monitors. Of these, 498 participated in the second-wave survey in June 2018 to confirm 1-month test–retest reliability. We examined the factorial validity of the BAT-J core symptoms (BAT-JC) and BAT-J secondary symptoms (BAT-JS), as well as their reliability (internal consistency and test–retest reliability) and construct validity. Factorial validity was examined using confirmatory factor analyses and exploratory structural equation modeling bifactor analyses. Convergent and discriminant validity were examined using multitrait–multimethod frameworks well as the average variance explained. Exploratory structural equation modeling bifactor solutions for the BAT-JC, BAT-JS, and BAT-J demonstrated the best fit to the data. They also indicated that the general factor accounted for over two-thirds of the common variance explained. Internal consistency and test–retest reliability were confirmed. Convergent and internal discriminant validity of the BAT-JC were confirmed vis-ȧ-vis burnout, as assessed with the Maslach Burnout Inventory – General Survey. Moreover, external discriminant validity of the BAT-J was demonstrated for work engagement and workaholism. Finally, both BAT scales showed significant positive relationships with job demands and turnover intention. All validity results were in line with the job demands–resources model. The results of the current study provide the first evidence for the BAT-J’s reliability and factorial and construct validity.

## Introduction

Burnout has become a matter of global concern for working people. It has been defined as “a state of exhaustion in which one is cynical about the value of one’s occupation and doubtful of one’s capacity to perform” (Maslach and Jackson, 1986, p. 20). Originally, burnout was exclusively identified with human services professionals; more recently, it has also been recognized in other occupations (Schaufeli et al., 2009c). In fact, the World Health Organization (2019) includes burnout in the recent 11th revision of the International Classification of Diseases as a global occupational phenomenon that influences health (World Health Organization, 2019); it does not, however, classify burnout as a medical condition but raises awareness of the importance of early assessment and appropriate treatment for burnout.

Research has confirmed that burnout predicts physical and psychological consequences, including cardiovascular diseases (Toppinen-Tanner et al., 2009; Toker et al., 2012), type 2 diabetes, musculoskeletal disorders (Melamed, 2009; Armon et al., 2010), depressive symptoms (Ahola and Hakanen, 2007; Armon et al., 2014; Bianchi et al., 2015), and insomnia (Armon et al., 2008). Furthermore, burnout predicts occupational consequences such as job dissatisfaction (Figueiredo-Ferraz et al., 2012; Lizano and Mor Barak, 2015), sickness-related absence (Borritz et al., 2006; Schaufeli et al., 2009a; Hallsten et al., 2011), and turnover intention (Geurts et al., 1998; Schaufeli and Bakker, 2004; Lin et al., 2013).

Most studies have used the Maslach Burnout Inventory (MBI; Maslach and Jackson, 1981), originally developed for human service workers, to assess burnout. Later, as the definition was expanded to include all occupations, a general version of the MBI – the MBI-General Survey (MBI-GS; Schaufeli et al., 1996) – was developed, consisting of three dimensions: exhaustion, cynicism, and professional efficacy.

Despite its popularity, researchers have pointed out various flaws of the MBI, related to conceptualization, psychometric shortcomings, and practical applicability. First, regarding conceptualization, the MBI does not include reduced cognitive functioning such as impaired attention, concentration, and working memory, which has been reported in recent studies (for an overview, see Deligkaris et al., 2014).

Second, the MBI suffers from psychometric shortcomings. Wheeler et al. (2011) conducted a meta-analysis of reliability coefficients for the subscales of the MBI and concluded: “personal accomplishment and depersonalization mean alpha estimates were well below recommended levels for high-stakes decisions, such as the diagnosis of burnout syndrome” (Wheeler et al., 2011, p. 213). Also, a study by De Beer and Bianchi (2019) reported results of confirmatory factor analysis (CFA) of the MBI, showing that a two-factor model composed of combined exhaustion and depersonalization/cynicism factor and a personal accomplishment factor showed the best fit to the data. Thus, the role of personal accomplishment/professional efficacy in burnout is debated. This is in line with the observation that, in many cases, only the exhaustion and/or depersonalization/cynicism subscales are used to assess burnout (Schaufeli and Taris, 2005).

Third, although burnout is recognized as an occupational disease in some European countries (Lastovkova et al., 2017), and there is a great need for a burnout measure that can be used in practice, the practical use of the MBI is rather poor. A key issue is that the MBI does not produce a single burnout score that can be dichotomized for screening employees who are or are not at risk for burnout. Tellingly, the MBI test manual states: “In general, each respondent’s scale scores should be calculated and interpreted separately. Note that responses to MBI items should not be combined to form a single ‘burnout score”’ (Maslach et al., 2017, p. 44).

In addition to the MBI, there are other burnout measures, such as the Copenhagen Burnout Inventory (Kristensen et al., 2005), Oldenburg Burnout Inventory (Demerouti et al., 2003), and the Shirom–Melamed Burnout Measure (Shirom and Melamed, 2006). However, these measures have weaknesses as well. For example, the Copenhagen Burnout Inventory assesses only exhaustion. Likewise, the Shirom–Melamed Burnout Measure consists of physical fatigue, emotional exhaustion, and cognitive weariness. Hence, these measures do not include withdrawal from work (cynicism/mental distance), which is a main feature of burnout together with exhaustion (Maslach et al., 2001; Schaufeli and Taris, 2005). Finally, the Oldenburg Burnout Inventory has two dimensions, including fatigue and disengagement (equivalent to cynicism). However, it does not include cognitive impairment, which has been reported as one of the characteristics of burnout (Deligkaris et al., 2014).

To overcome the flaws of the MBI and other burnout measures, Schaufeli et al. (2019) developed a more comprehensive conceptualization of burnout and introduced a new instrument for assessing it, the Burnout Assessment Tool (BAT). The BAT is based on a combination of deductive and inductive approaches. The deductive approach comprises a theoretical description of burnout as a primary work-related syndrome of exhaustion and mental distancing (Schaufeli and Taris, 2005). Moreover, 13 burnout questionnaires were analyzed to examine which dimensions (and items) they included. Content analyses revealed that all 13 questionnaires contained an exhaustion dimension; three only included exhaustion, two included exhaustion and secondary symptoms, one included only secondary symptoms, and the remaining seven were multidimensional and included a mental distance dimension. Hence, the analyses revealed that exhaustion and mental distance were common core components of all multidimensional burnout measures.

The inductive approach included in-depth, face-to-face, semi-structured interviews. The interviews aimed to reconceptualize burnout as it appears in today’s working environments, which has changed since the introduction of the MBI (Maslach and Jackson, 1981) 40 years ago. Interviews were conducted with 49 Flemish and Dutch professionals who handle individuals with burnout on a daily basis. To identify typical symptoms of burnout, interviewees were asked to describe a typical burnout case, the specific symptoms and causes of burnout, and their own definition of burnout. Next, interviewees ranked the symptoms they mentioned following the importance of assessing burnout; the professionals identified 260 symptoms. These qualitative data were categorized into seven dimensions after two rounds of content analysis: exhaustion, mental distance, emotional impairment, cognitive impairment, depressed mood, psychological distress, and psychosomatic complaints. The seven dimensions were clustered into core dimensions and secondary dimensions based on the theorizing of Schaufeli and Taris (2005) and the interview results. The core dimensions were exhaustion, mental distance, emotional impairment, and cognitive impairment. Exhaustion was the most obvious symptom that was mentioned by all interviewees, but it is not a sufficient condition for burnout. Interviewees also pointed to mental distance, emotional impairment, and cognitive impairment, which appear along with exhaustion in those who suffer from burnout. Additionally, three secondary dimensions were identified: depressed mood, psychological distress, and psychosomatic complaints. Because these symptoms are atypical and also appear with other disorders such as mood disorder, anxiety disorder, and cancer, they were considered secondary.

From this work, burnout was reconceptualized as “a work-related state of exhaustion that occurs among employees, which is characterized by extreme tiredness, reduced ability to regulate cognitive and emotional processes, and mental distancing. These four core dimensions of burnout are accompanied by depressed mood as well as by non-specific psychological and psychosomatic complaints” (Schaufeli et al., 2019, p. 29).

Along with the new definition, a new instrument for assessing burnout, Burnout Assessment Tool (BAT) was developed. The BAT assesses four core symptoms, referred to as BAT-C (exhaustion, mental distance, emotional impairment, and cognitive impairment), and two secondary symptoms, referred to as BAT-S (psychological distress and psychosomatic complaints). The remaining secondary dimension, depressed mood, was not included in the new burnout instrument because other well-validated depression questionnaires, such as the depression subscale of the 4-Dimensional Symptom Questionnaire (Terluin et al., 2006), are available.^1^

Although burnout is not recognized as a formal diagnosis in Japan, it is still crucial to identify employees with burnout and provide appropriate prevention and treatment because burnout has adverse effects on both employees’ health and organizational effectiveness. Because there is no established procedure to assess burnout in Japan (Kitaoka et al., 2011), it is of vital importance to validate an instrument that can be used as a screening tool for burnout in occupational health settings. In this regard, the validation of the Japanese version of BAT (BAT-J) is a necessary first step. Therefore, the current study aimed to validate the BAT-J (consisting of BAT-JC for core symptoms and BAT-JS for secondary symptoms).

We analyzed the BAT-J in three steps: first, factorial validity was assessed using CFA and exploratory structural equation modeling (ESEM) bifactor analysis; second, the reliability was assessed using internal consistency and test–retest reliability; and third, construct validity was assessed by evaluating convergent and discriminant validity. For the convergent and internal discriminant validity, we compared the BAT-J with the MBI-GS, using a multitrait–multimethod (MTMM) model (Campbell and Fiske, 1959). For external discriminant validity, we compared the average variance explained (AVE; Fornell and Lacker, 1981) of the BAT-JC and BAT-JS with work engagement and workaholism, where work engagement was defined as a positive, fulfilling, work-related state of mind characterized by vigor, dedication, and absorption (Schaufeli et al., 2002), and workaholism was defined as the uncontrollable inner need to work extremely hard (Schaufeli et al., 2009b). Workaholism includes both behavioral (excessive working) and cognitive (compulsive working) dimensions. Previous studies confirmed that burnout and work engagement are negatively related, whereas burnout and workaholism are positively related (Schaufeli and Bakker, 2010). Further, the concepts can be discriminated from each other (Schaufeli et al., 2008). Also, we assessed the construct validity of the BAT-J by adopting the conceptual framework of the job demands–resources (JD-R) model (Demerouti et al., 2001). The core idea of the JD-R model is that high job demands produce high levels of stress and subsequent health impairment, whereas high job resources lead to high levels of motivation and subsequent superior job performance. Specifically, we examined the association of the BAT-J with potential antecedents (i.e., job demands) and potential consequences (i.e., performance). Previous studies confirmed that job demands are consistently found to be antecedents of burnout (Schaufeli and Salanova, 2014), and burnout predicts organizational outcomes (Geurts et al., 1998; Schaufeli and Bakker, 2004).

## Materials and Methods

### Translation

First, the English version of the BAT was translated into Japanese by the current study authors (KS and AS). Next, a bilingual (Japanese and English) psychologist, who had not read the original items, conducted back-translation into English. We compared the original English and the back-translated versions (WS) and harmonized them. Further, we conducted cognitive interviews with corporate employees and finalized the preliminary Japanese version after some corrections for words, meanings, and item content by the authors (KS, AS, and HT).

### Participants

The current study was based on two waves of surveys, using the registered monitors of a survey company. The first survey was conducted in May 2018, and 22,249 employed monitors were invited to participate. Participants were equally allocated by sex and generation. Because of budgetary constraints, recruitment stopped after the number of participants exceeded 1,420. Data from 982 respondents who met the inclusion criteria (full-time employment and under 64 years old) were used in the analyses. The second-wave survey was conducted in June 2018 to confirm test–retest reliability. Again, because of budget constraints, 498 of the original respondents were invited to participate. Of these, 485 completed the questionnaire, yielding a response rate of 97.4% for the second survey. Table 1 shows the respondents’ characteristics: mean age was 39.8 years (*SD* = 11.3); 51.0% were male; 50.5% were married or cohabiting; 50.3% had a university degree; 83.2% were white-collar workers, and 11.1% were shift workers; the mean working time per week was 40.3 h (*SD* = 18.7).

**TABLE 1:** Demographic characteristics of the study participants (*N* = 982).

	**n (%)**	**Mean (*SD*)**
Age (year)		39.8 (11.3)
**Gender**		
Men	501 (51.0)	
Women	481 (49.0)	
**Marriage**		
Yes (including co-habitant)	496 (50.5)	
No	486 (49.5)	
**Education**		
Collage or lower	488 (49.7)	
University or higher	494 (50.3)	
**Occupation**		
White collar	817 (83.2)	
Blue collar	165 (16.8)	
**Shift work**		
No	873 (88.9)	
Yes	109 (11.1)	
Working hours/week		40.3 (18.7)

#### Ethical Considerations

The Ethics Review Board of Toyo University approved the procedures before starting the study. Participants had the option of not responding to any part of the questionnaire at any time and to discontinue the survey at any point. Participants’ consent was confirmed based on their completion of the questionnaire.

### Measures

#### Burnout

Burnout was assessed with a preliminary version of BAT-J and the Japanese version of MBI-GS (Maslach and Jackson, 1986; Kitaoka-Higashiguchi et al., 2004). The BAT-J consists of two components: BAT-JC and BAT-JS. The BAT-JC includes 23 items, measuring four core symptoms of burnout: exhaustion (eight items; e.g., *“*At work, I feel mentally exhausted,” α = 0.93), mental distance (five items; e.g., “I struggle to find any enthusiasm for my work,” α = 0.86), emotional impairment (five items; e.g., “At work, I feel unable to control my emotions,” α = 0.91), and cognitive impairment (five items; e.g., “At work, I have trouble staying focused,” α = 0.93). The BAT-JS includes 10 items measuring secondary symptoms: psychological distress (five items; e.g., “I have trouble falling or staying asleep,” α = 0.89) and psychosomatic complaints (five items; e.g., “I suffer from palpitations or chest pain,” α = 0.87). All items were scored on a five-point Likert scale ranging from 1 (never) to 5 (always). Responses were summed and averaged for each subscale. The MBI-GS was used to confirm the BAT-J’s convergent and discriminant validity. The MBI-GS subscales include exhaustion (five items; e.g., “I feel tired when I get up in the morning and have to face another day on the job,” α = 0.94), cynicism (five items; e.g., “I have become more cynical about whether my work contributes anything,” α = 0.78), and professional efficacy (six reverse-scored items; e.g., “I feel I am making an effective contribution to what this organization does,” α = 0.66). All items were scored on a seven-point Likert scale ranging from 0 (never) to 6 (every day). Responses were summed and averaged for each subscale.

#### Work-Related Well-Being

Work engagement was assessed with the short form of the Utrecht Work Engagement Scale (Schaufeli et al., 2002), which has been validated in Japan (Shimazu et al., 2008). The scale includes three subscales: vigor (three items; e.g., “At my job, I feel strong and vigorous,” α = 0.90), dedication (three items; e.g., “I am enthusiastic about my job,” α = 0.88), and absorption (three items; e.g., “I am immersed in my work,” α = 0.90). All items were scored on a seven-point Likert scale ranging from 0 (never) to 6 (always). Responses were summed and averaged for each subscale, as recommended by Schaufeli et al. (2006).

Workaholism was assessed with the Dutch Work Addiction Scale (Schaufeli et al., 2009d), which includes two subscales: working excessively (five items; e.g., “I seem to be in a hurry and racing against the clock,” α = 0.81) and working compulsively (five items; e.g., “I feel obliged to work hard, even when it’s not enjoyable,” α = 0.79). All items were scored on a four-point Likert scale from 1 (almost never) to 4 (almost always). Responses were summed and averaged for each subscale.

#### Potential Antecedents

Quantitative and qualitative job demands were assessed using subscales of the Brief Job Stress Questionnaire (Shimomitsu et al., 2000), whereas emotional demands were assessed using a subscale of the new version of the Brief Job Stress Questionnaire (Inoue et al., 2014). Sample items of each subscale include quantitative job demands (three items; e.g., “I have an extremely large amount of work to do,” α = 0.80); qualitative job demands (three items; e.g., “My job is difficult in that it requires a high level of knowledge and technical skill,” α = 0.74); and emotional demands (three items; e.g., “My job puts emotional burden on me,” α = 0.87). All items were scored on a four-point Likert scale from 1 (disagree) to 4 (agree). Responses were summed and averaged for each subscale.

#### Potential Consequences

We assessed turnover intention as a potential consequence and used three items developed by Geurts et al. (1998), translated into Japanese, and validated (Tsuno et al., 2018). Originally, this scale consisted of four items – three items were negatively worded, and one was positively worded and reverse-scored. When four items were used, Cronbach’s α was low (0.46). Therefore, we excluded the positively worded item and used the remaining three items, and the Cronbach’s α increased to 0.86. Participants were asked to rate the extent to which they felt like leaving their organization over the last month (e.g., “I consider my decision to work for this employer as an obvious mistake,” α = 0.86). Items were scored on a five-point Likert scale ranging from 1 (completely agree) to 5 (completely disagree). Responses for the three items were summed and averaged.

### Data Analyses

#### Factorial Validity

We assessed the factorial validity of the BAT-JC, BAT-JS, and BAT-J using CFA and ESEM bifactor analysis using Mplus 8.0, based on the robust maximum likelihood estimator. We compared four models. First, a correlated CFA model was tested to examine the correlations among the latent factors. Second, a second-order CFA model was tested. This model was based on the assumption that burnout is a syndrome comprising a set of related symptoms referring to one underlying psychological condition (i.e., burnout). Another reason for examining a second-order model was that we needed to confirm whether the BAT could produce a single score. Third, a CFA bifactor model was tested. Second-order and bifactor models are similar because both examine the presence of global and specific factors corresponding to multiple items. In second-order CFA, each item is assumed to load on its particular subscale (a first-order factor), and each first-order factor is assumed to load on a second-order factor (Rindskopf and Tedd, 1988). On the other hand, a bifactor model directly tests whether a global construct exists as a common dimension of all items and multiple more specific facets, defined by the items belonging to the facets, coexist as remaining parts that are not explained by the global factor (Morin et al., 2016). Finally, the ESEM bifactor model analysis was conducted based on oblique target rotation (Asparouhov and Muthén, 2009). In the multidimensional scale, factors are usually related to each other, and it is assumed that items belonging to each factor have some association with other factors as well. However, in CFA, each item is forced to load on one target factor, and that causes inflation of the estimated factor correlations (Morin et al., 2016). ESEM provides a solution for this problem by allowing the cross-loading of items on non-target factors (Marsh et al., 2014).

For the BAT-JC, model C1 was a correlated four-factor CFA model where four different components (exhaustion, mental distance, emotional impairment, and cognitive impairment) were correlated. Model C2 was a second-order CFA model assuming that burnout is a syndrome comprising the four core dimensions mentioned earlier. Model C3 was a CFA bifactor model where each item was related to the expected specific core dimension and the global factor (burnout). Model C4 was an ESEM bifactor model where all items of BAT-JC were allowed to load on a general factor (burnout), and each item was simultaneously allowed to load on the specific target factor, as well as non-target factors.

For the BAT-JS, model S1 was a correlated two-factor CFA model where two different components (psychological distress and psychosomatic complaints) are correlated. Model S2 was a second-order CFA model assuming that secondary burnout symptoms comprise two dimensions. Model S3 was a CFA bifactor model where each item was related to the expected specific factor and a global factor (secondary burnout symptom). Model S4 was an ESEM bifactor model where all items of the BAT-JS were allowed to load on a general factor (secondary burnout symptoms), and each item was allowed to load on a specific target factor (psychological and psychosomatic symptoms) as well as a non-target factor.

The BAT-J, model J1 was a correlated six-factor CFA model where all six factors of the BAT-JC and the BAT-JS (exhaustion, mental distance, emotional impairment, cognitive impairment, psychological distress, and psychosomatic complaints) were correlated. Model J2 was a second-order CFA model where six components were first-order factors, and burnout was the higher-order factor. Model J3 was a CFA bifactor model where each item was related to the expected specific target factor and a global factor (burnout). Finally, Model J4 was an ESEM bifactor model where all items of the BAT were allowed to load on a general factor (burnout), and each item was allowed to load on a specific factor with cross-loadings on non-target factors.

The mean item loadings on the general factor, specific factors, and the explained common variance (ECV), an index of the proportion of common variance extracted explained by the general factor (Rodriguez et al., 2016), were calculated. Higher ECV values showed a strong general factor, suggesting the measurement was unidimensional, even if multiple factors were involved (Reise, 2012). Item level ECV (I-ECV) and specific factor level ECV (S-ECV) were also calculated. Following Hu and Bentler (1995), model fit was assessed using a combination of fit indices: chi-square (χ^2^), Tucker–Lewis index (TLI), comparative fit index (CFI), and root mean square error of approximation (RMSEA). The model fit was evaluated using the following criteria: both TLI and CFI exceeded at least 0.90 but preferably 0.95 (Hu and Bentler, 1995), and RMSEA was < 0.08 (Byrne, 2016, p. 98). We also used several information criteria, including Akaike Information Criterion (AIC), the constant AIC, the Bayesian Information Criterion (BIC), and the sample-size adjusted BIC to compare the alternative models, with lower values being a better fit.

#### Reliability

We evaluated the scale’s reliability by assessing the internal consistency, based on the score of Cronbach’s α of each subscale and the composite BAT-JC and BAT-JS scales. We also assessed the test–retest reliability of the BAT-J with the stability coefficients of the scores between the first and second surveys.

#### Construct Validity

**FIGURE 1:**
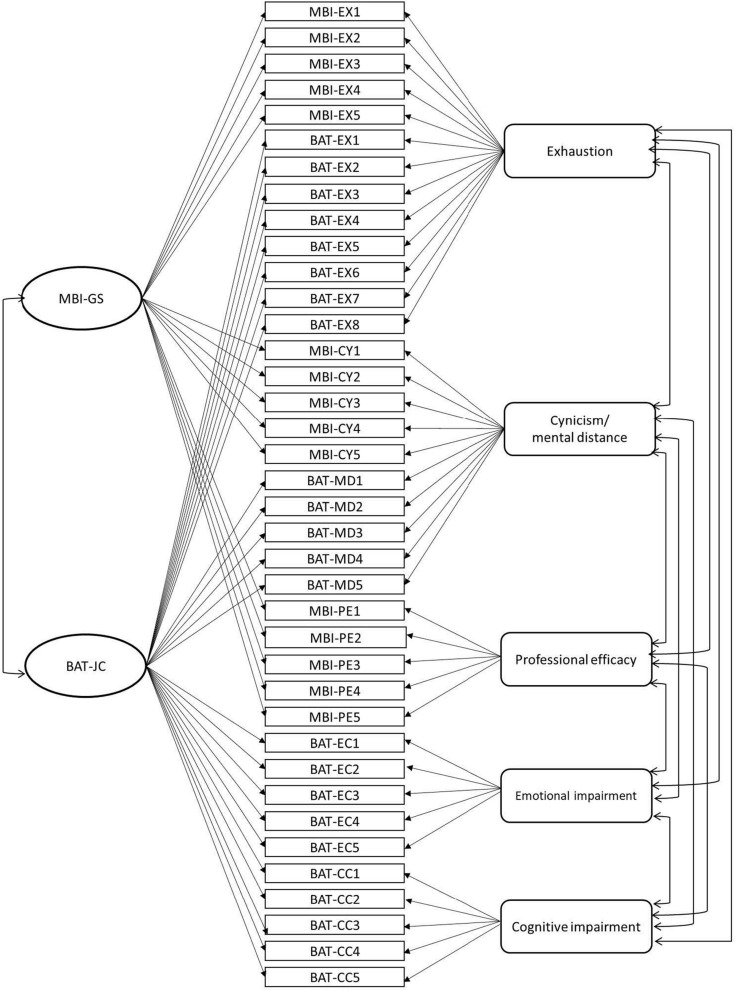
Graphical presentation of the MTMM model, including two method factors (MBI-GS and BAT-JC) and five trait factors (exhaustion, cynicism/mental distance, professional efficacy, emotional impairment, cognitive impairment). MBI-EX1, item 1 of the exhaustion subscale of the MBI-GS; MBI-CY1, item 1 of the cynicism subscale of the MBI-GS; MBI-PE1, item 1 of the professional efficacy subscale of the MBI-GS; BAT-EX1, item 1 of the exhaustion subscale of the BAT-JC; BAT-MD1, item 1 of the mental distance subscale of the BAT-JC; BAT-EC1, item 1 of the emotional impairment subscale of the BAT-JC; BAT-CC1, item 1 of the cognitive impairment subscale of the BAT-JC.

We assessed construct validity in terms of convergent and internal discriminant validity using the MTMM model (Campbell and Fiske, 1959). MTMM is an approach for examining convergent and discriminant validity by confirming how a measure relates to other measures. Figure 1 shows a graphical presentation of the MTMM model, including two-method factors (measures: the BAT-JC and the MBI-GS) and five-trait factors (constructs: exhaustion, cynicism/mental distance, professional efficacy, emotional impairment, and cognitive impairment). Because the MBI-GS does not measure secondary symptoms of burnout, we focused on the BAT-JC in this analysis. We followed the guidelines by Widaman (1985) and compared four models.

Model 1, the correlated traits–correlated methods (CT-CM) model, was based on the assumption that the structure of the data is determined by both trait factors (constructs) and method factors (measures). The CT-CM model served as the baseline against which all other MTMM models were compared. It was the least restrictive model in which all trait factors were free to correlate with each other, and both method factors (the BAT-JC and the MBI-GS) were free to correlate with each other. Trait and method factors were not allowed to correlate with each other.

Model 2, the no traits-correlated methods (NT-CM) model, was based on the assumption that there is no correlation between trait factors, and the structure of the data could only be described by methods (the BAT-JC and the MBI-GS). To evaluate convergent validity, we compared models 1 and 2. If model 1 fits the data better than model 2, it would suggest that the BAT-JC and the MBI-GS are independent of each other, but some traits (constructs) are correlated.

Model 3, the perfectly correlated traits-correlated methods (PCT-CM) model, assumed that the traits (constructs) correlated perfectly (fixed to 1), and the methods (measures) were freely correlated.

Model 4, the correlated traits-perfectly correlated methods (CT-PCM) model, assumed that the trait-factors (constructs) were freely correlated, and the methods (measures) correlated perfectly (fixed to 1). To test the discriminant validity, we compared model 1 with 3 in terms of traits (constructs) and model 1 with 4 in terms of method (measures). If the BAT-JC was distinct from the MBI-GS, traits and methods would be correlated but not perfectly, and if so, model 1 was assumed to have a better fit than models 3 and 4.

We assessed the external discriminant validity of the BAT concerning work engagement and workaholism using Average Variance Explained (AVE). In the current study, the AVE of the BAT-JC and BAT-JS should be greater than their squared correlations (*R*^2^) with work engagement and workaholism. To test this assumption, a general CFA model was evaluated in which the BAT-JC, BAT-JS, work engagement, and workaholism were correlated with each other.

In addition, based on the JD-R model, we assessed the construct validity of the BAT in relation to possible antecedents (quantitative and qualitative job demands and emotional demands) and consequences (turnover intention), using structural equation modeling techniques. Goodness of fit, χ^2^, TLI, CFI, and RMSEA were used to evaluate the models. The level of significance was 0.05 (two-tailed).

We used Mplus 8.0 for the CFA and ESEM bifactor analyses. We used IBM SPSS Statistics for Windows, Version 25, and Amos 24 software to analyze MTMM, AVE, and the relations of the BAT with potential antecedents and consequences.

## Results

### Factorial Validity

Table 2 shows the goodness-of-fit indices and information criteria of each model. For the BAT-JC, model C1, the correlated four-factor CFA, demonstrated good fit (CFI = 0.93, TLI = 0.92, RMSEA = 0.06). Correlations among the latent factors ranged from 0.65 to 0.83. Model C2, the second-order CFA, and model C3, the bifactor CFA, also demonstrated good fit (model C2; CFI = 0.93, TLI = 0.92, RMSEA = 0.06, model C3; CFI = 0.95, TLI = 0.93, RMSEA = 0.06). Model C4, the bifactor ESEM, showed best fit to the data (CFI = 0.98, TLI = 0.96, RMSEA = 0.04) and was a better presentation than the other three models based on lower scores on the information criteria.

**TABLE 2:** Model fit indices and information criteria for BAT-JC, BAT-JS, and BAT-J (*N* = 982).

**Model**	**χ^2^**	***p***	***df***	**RMSEA**	**(90%CI)**	**CFI**	**TLI**	**AIC**	**BIC**	**CAIC**	**ABIC**
**BAT-JC**											
Correlated four-factor CFA	1054.02	0.000	224	0.06	[0.06 0.07]	0.93	0.92	50,966	51,332	51,115	51,094
Second-order CFA	1076.94	0.000	226	0.06	[0.06 0.07]	0.93	0.92	51,000	51,357	51,146	51,125
Bifactor CFA	831.73	0.000	208	0.06	[0.05 0.06]	0.95	0.93	50,626	51,071	50,807	50,782
Bifactor ESEM	428.08	0.000	148	0.04	[0.04 0.05]	0.98	0.96	50,130	50,869	50,431	50,389
**BAT-JS**											
Correlated two-factor CFA	268.83	0.000	34	0.08	[0.08 0.09]	0.96	0.95	25,249	25,401	25,311	25,303
Second-order CFA	268.83	0.000	34	0.08	[0.08 0.09]	0.96	0.95	25,249	25,401	25,311	25,303
Bifactor CFA	83.95	0.000	25	0.05	[0.04 0.06]	0.98	0.97	25,127	25,323	25,207	25,196
Bifactor ESEM	28.69	0.052	18	0.03	[0.00 0.04]	1.00	0.99	25,055	25,284	25,148	25,135
**BAT-J**											
Correlated six-factor CFA	1774.08	0.000	480	0.05	[0.05 0.06]	0.92	0.92	75,166	75,723	75,393	75,361
Second-order CFA	1963.17	0.000	489	0.06	[0.05 0.06]	0.91	0.91	75,433	75,946	75,642	75,613
Bifactor CFA	1683.85	0.000	462	0.05	[0.05 0.06]	0.93	0.92	75,040	75,685	75,303	75,266
Bifactor ESEM	706.05	0.000	318	0.04	[0.03 0.04]	0.98	0.96	73,863	75,212	74,412	74,336

*TLI, Tucker–Lewis index; CFI, comparative fit index; RMSEA, root mean square error of approximation; χ^2^, *chi-square; df, degree of freedom; p, p-value; AIC, Akaike information criterion; BIC, Bayesian information criterion; CAIC, constant AIC; ABIC, sample-size-adjusted BIC.**

For the BAT-JS, model S1, the correlated two-factor CFA, and model S2, the second-order CFA, both showed good fit (TLI = 0.95, CFI = 0.96, RMSEA = 0.08). For model S1, correlations among the two latent factors were 0.84. Model S3, the bifactor CFA, demonstrated better fit (CFI = 0.98, TLI = 0.97, RMSEA = 0.05). Model S4, the bifactor ESEM, showed the best fit for the data (CFI = 1.0, TLI = 0.99, RMSEA = 0.03) with the lower scores on the information criteria compared to Model S1, S2, and S3.

Finally, for the BAT-J, model J1 (correlated six-factor CFA), J2 (second-order CFA), and J3 (bifactor CFA) showed good fit (model J1: CFI = 0.92, TLI = 0.92, RMSEA = 0.05; model J2: CFI = 0.91, TLI = 0.91, RMSEA = 0.06; model J3: CFI = 0.93, TLI = 0.92, RMSEA = 0.05). For model J1, correlations among the latent factors ranged from 0.31 to 0.62. The four core dimensions were more strongly related with each other (ranging from 0.43 to 0.72) than with the two secondary dimensions (ranging from 0.41 to 0.62). Model J4, the bifactor ESEM, demonstrated the best fit to the data (CFI = 0.98, TLI = 0.96, RMSEA = 0.04) with the lower scores on the information criteria compared with other models. Thus, the bifactor ESEM provided the best presentation of the data for the BAT-JC, BAT-JS, and BAT-J.

Table 3 shows the standardized factor loadings of the bifactor ESEM for the BAT-JC, BAT-JS, and BAT-J. All items of the BAT-JC loaded substantially on the general factor (|λ| = 0.39–0.90). The ECV index showed that the general factor accounted for 70% of the common variance extracted. Regarding factor loadings on the specific factors, target loadings on exhaustion (|λ| = 0.40–0.61) and cognitive impairment (|λ| = 0.32–0.46) were all significant. Target loadings on mental distance and emotional impairment were low to moderate (|λ| = 0.05–0.55 and 0.02–0.50, respectively); four out of five possible loadings for mental distance and three out of the five possible loadings for emotional impairment were significant.

**TABLE 3:** Standardized factor loading for bifactor exploratory equation modeling analysis of BAT-JC, BAT-JS, and BAT-J (*N* = 982).

			**General factor**	**Bifactors**								
**Scale**	**Subscale**	**Item**	**BAT-JC**	**Exhaustion**	**Mental distance**	**Emotional impairment**	**Cognitive impairment**	**I-ECV**	**S-ECV**	**ECV**
BAT-JC	Exhaustion	1	0.61***	0.53***	0.03	0.02	–0.04	0.57	0.16	0.70
		2	0.39***	0.47***	−0.22***	–0.04	–0.01	0.36		
		3	0.61***	0.61***	0.02	–0.03	0.01	0.50		
		4	0.60***	0.61***	–0.03	0.00	–0.03	0.49		
		5	0.63***	0.52***	0.11***	0.03	0.05	0.58		
		6	0.64***	0.40***	0.19***	0.00	0.06	0.67		
		7	0.53***	0.57***	–0.01	0.04	0.00	0.46		
		8	0.63***	0.61***	0.03	–0.01	–0.04	0.51		
	Mental distance	9	0.61***	0.30***	**0.05**	–0.01	0.04	0.80	0.05	
		10	0.65***	0.15***	0.31***	–0.03	0.06	0.77		
		11	0.74***	0.11**	0.32***	0.02	–0.05	0.83		
		12	0.66***	−0.06*	0.55***	0.00	0.05	0.59		
		13	0.65***	–0.03	0.41***	0.02	0.02	0.71		
	Emotional impairment	14	0.79***	–0.03	0.11***	0.28***	0.03	0.87	0.03	
		15	0.78***	–0.05	–0.05	0.50**	–0.02	0.71		
		16	0.69***	0.09*	–0.03	0.37***	–0.02	0.77		
		17	0.81***	−0.20***	–0.07	−**0.02**	–0.02	0.94		
		18	0.90***	−0.16**	−0.19***	−**0.05**	−0.08***	0.92		
	Cognitive impairment	19	0.77***	0.00	0.05	–0.01	0.35***	0.82	0.05	
		20	0.80***	0.07**	0.03	–0.01	0.34***	0.84		
		21	0.73***	0.02	–0.02	0.01	0.44***	0.73		
		22	0.75***	−0.05*	0.08**	0.02	0.46***	0.72		
		23	0.74***	–0.04	–0.04	–0.03	0.32***	0.83		

			**General factor**	**Bifactors**								
**Scale**	**Subscale**	**Item**	**BAT-JS**	**Psychological distress**		**Psychosomatic complaints**		**I-ECV**	**S-ECV**	**ECV**

BAT-JS	Psychological distress	24	0.75***	**0**.37***		−0.16		0.78	0.15	0.78
		25	0.82***	**0**.66***		0.13		0.60		
		26	0.69***	**0**.69***		0.36***		0.44		
		27	0.91***	**0**.41***		0.58***		0.62		
		28	0.83***	**0**.32***		0.15***		0.84		
	Psychosomatic complaints	29	0.84***	−0.12***		**0.04**		0.98	0.07	
		30	0.91***	0.01		**0.02**		1.00		
		31	0.89***	−0.01		**0**.20***		0.95		
		32	0.81***	0.10***		−0.15***		0.95		
		33	0.86***	0.05		−**0.03**		1.00		

			**General factor**	**Bifactors**								
**Scale**	**Subscale**	**Item**	**BAT-J**	**Exhaustion**	**Mental distance**	**Emotional impairment**	**Cognitive impairment**	**Psychological distress**	**Psychosomatic complaints**	**I-ECV**	**S-ECV**	**ECV**

BAT-J	Exhaustion	1	0.62***	0.52***	0.05	0.04	–0.02	0.09**	-0.08*	0.57	0.11	0.69
		2	0.41***	0.45***	−0.23***	–0.03	–0.01	0.01	-0.09*	0.39		
		3	0.64***	0.57***	0.02	–0.05	–0.02	-0.01	0.04	0.55		
		4	0.62***	0.59***	–0.02	0.01	–0.03	-0.01	0.05	0.52		
		5	0.66***	0.47***	0.11***	–0.01	0.03	0.07	0.05	0.64		
		6	0.67***	0.36***	0.20***	–0.02	0.03	0.05	0.03	0.72		
		7	0.55***	0.55***	0.00	0.04	0.00	0.00	0.05	0.49		
		8	0.66***	0.58***	0.02	–0.02	−0.06*	0.03	0.00	0.56		
	Mental distance	9	0.61***	0.28***	**0.05**	0.01	0.03	0.01	-0.07*	0.81	0.04	
		10	0.63***	0.16***	0.34***	0.00	0.08*	-0.07	-0.06*	0.72		
		11	0.73***	0.10**	0.34***	0.04	–0.04	0.01	-0.12***	0.79		
		12	0.63***	−0.06*	0.58***	0.02	0.08**	-0.04	-0.07**	0.53		
		13	0.62***	–0.03	0.44***	0.06	0.04	-0.06	0.01	0.65		
	Emotional impairment	14	0.77***	–0.03	0.15***	0.31***	0.04	-0.03	-0.06*	0.82	0.03	
		15	0.73***	–0.02	0.03	0.55***	0.04	0.00	-0.03	0.64		
		16	0.65***	0.10***	0.03	0.45***	0.03	0.10**	-0.03	0.66		
		17	0.83***	−0.25***	−0.07*	**0.06**	–0.07	-0.15***	-0.07*	0.87		
		18	0.84***	−0.13***	−0.10***	0.16*	0.01	-0.10***	-0.08**	0.91		
	Cognitive impairment	19	0.76***	–0.01	0.08*	0.05	0.38***	-0.04	0.00	0.79	0.03	
		20	0.80***	0.05*	0.06*	0.02	0.35***	0.01	-0.02	0.83		
		21	0.74***	–0.02	–0.01	0.00	0.41***	0.00	-0.05	0.76		
		22	0.76***	−0.08***	0.09***	0.02	0.44***	0.00	0.00	0.74		
		23	0.75***	−0.07**	–0.02	0.01	0.31***	-0.03	0.02	0.84		
	Psychological distress	24	0.63***	0.03	–0.06	–0.04	0.01	**0**.24***	0.16***	0.82	0.03	
		25	0.74***	0.02	–0.06	0.01	–0.04	**0**.42***	0.07*	0.75		
		26	0.68***	0.27***	–0.02	0.07*	0.01	**0**.45***	0.05	0.62		
		27	0.82***	−0.09**	−0.08*	−0.07*	–0.05	**0**.28***	0.12**	0.86		
		28	0.69***	–0.01	–0.03	0.00	0.03	**0**.21***	0.19***	0.85		
	Psychosomatic complaints	29	0.68***	−0.19***	–0.03	−0.10*	–0.06	-0.10**	**0**.33***	0.74	0.05	
		30	0.64***	−0.07**	–0.01	–0.05	0.00	0.11***	**0**.47***	0.63		
		31	0.60***	0.03	−0.06*	−0.06*	–0.01	0.08**	**0**.51***	0.57		
		32	0.50***	0.18***	–0.04	0.06	0.04	0.13***	**0**.50***	0.45		
		33	0.66***	0.03	−0.07*	–0.02	–0.03	0.08*	**0**.46***	0.66		

ECV, explained common variance; I-ECV, item level ECV; S-ECV, specific ECV. Target factor loadings are in bold. ***p < 0.001, **p < 0.01, *p < 0.05.

For the BAT-JS, all items loaded substantially on the general factor (|λ| = 0.69–0.91), and the ECV index showed that the general factor accounted for 78% of the common variance extracted. Regarding factor loadings on the specific factors, target loadings on psychological distress (|λ| = 0.32–0.69) were all significant. Target loadings on psychosomatic complaints were low (|λ| = 0.02–0.20), and only two of five possible loadings were significant.

For the BAT-J, all items loaded substantially on the general factor (|λ| = 0.41–0.84), and the ECV index showed that the general factor accounted for 69% of the common variance extracted. Regarding factor loadings on the specific factors, target loadings on exhaustion (|λ| = 0.36–0.59), cognitive impairment (|λ| = 0.31–0.44), psychological distress (|λ| = 0.21–0.45), and psychosomatic complaints (|λ| = 0.33–0.51) were moderate, and all of the possible loadings were significant. Target loading on mental distance (|λ| = 0.05–0.58) and emotional impairment (|λ| = 0.06–0.55) were low to moderate; four out of five possible loadings for each factor were significant.

### Reliability

Internal consistencies are shown in Table 4. The four subscales of BAT-JC and the two subscales of BAT-JS show a high level of internal consistency. Cronbach’s α for all subscales, except mental distance, exceeded 0.90; for the composite BAT-JC scale, Cronbach’s α was 0.96. For the BAT-JS, Cronbach’s α for both subscales ranged from 0.87 to 0.89 and was 0.92 for the composite BAT-JS. As for the test–retest reliability, the BAT-JC and BAT-JS correlated 0.64 and 0.71 (*p* < 0.001), respectively, across a time interval of 1 month.

**TABLE 4:** Means, standard deviations, internal consistencies (Cronbach’s α) and correlations of the variables used in the study (*N* = 982).

		**Range**	**Mean**	***SD***	**α**	**1**	**2**	**3**	**4**	**5**	**6**	**7**	**8**	**9**
1	BAT-JC	1–5	2.58	0.79	0.96									
2	Exhaustion	1–5	2.95	0.89	0.93	0.87***								
3	Mental distance	1–5	2.44	0.88	0.86	0.88***	0.67***							
4	Emotional impairment	1–5	2.35	0.93	0.91	0.87***	0.61***	0.74***						
5	Cognitive impairment	1–5	2.37	0.94	0.93	0.88***	0.61***	0.74***	0.79***					
6	BAT-JS	1–5	2.38	0.89	0.92	0.78***	0.66***	0.63***	0.72***	0.73***				
7	Psychological distress	1–5	2.52	1.00	0.89	0.79***	0.68***	0.64***	0.72***	0.72***	0.94***			
8	Psychosomatic complaints	1–5	2.28	0.94	0.87	0.67***	0.56***	0.54***	0.61***	0.63***	0.93***	0.75***		
9	MBI-GS	0–6	2.44	1.05	0.90	0.73***	0.72***	0.62***	0.59***	0.57***	0.64***	0.63***	0.57***	
10	Exhaustion	0–6	2.54	1.58	0.94	0.80***	0.85***	0.66***	0.60***	0.60***	0.69***	0.69***	0.59***	0.87***
11	Cynicism	0–6	2.37	1.20	0.78	0.69***	0.61***	0.64***	0.58***	0.58***	0.59***	0.59***	0.51***	0.90***
12	Reduced professional efficacy	0–6	2.42	1.01	0.72	0.29***	0.29***	0.20***	0.29***	0.22***	0.30***	0.27***	0.29***	0.74***
13	Work engagement	0–6	2.32	1.24	0.95	−0.31***	−0.26***	−0.38***	−0.21***	−0.24***	−0.19***	−0.22***	−0.13***	−0.13***
14	Vigor	0–6	2.18	1.28	0.90	−0.29***	−0.28***	−0.34***	−0.19***	−0.21***	−0.19***	−0.23***	−0.13***	−0.14***
15	Dedication	0–6	2.54	1.33	0.88	−0.33***	−0.23***	−0.42***	−0.24***	−0.27***	−0.20***	−0.22***	−0.14***	−0.12***
16	Absorption	0–6	2.23	1.36	0.90	−0.25***	−0.21***	−0.32***	−0.15***	−0.20***	−0.14***	−0.17***	–0.09	−0.10***
17	Workaholism	1–4	2.00	0.63	0.88	0.41***	0.42***	0.29***	0.38***	0.34***	0.38***	0.38***	0.32***	0.42***
18	Working excessively	1–4	2.04	0.71	0.81	0.41***	0.44***	0.26***	0.36***	0.32***	0.36***	0.37***	0.30***	0.43***
19	Working compulsively	1–4	1.96	0.66	0.79	0.36***	0.33***	0.27***	0.34***	0.30***	0.34***	0.34***	0.30***	0.36***
20	Quantitative job demand	1–4	2.58	0.73	0.80	0.37***	0.46***	0.22***	0.29***	0.25***	0.27***	0.28***	0.22***	0.30***
21	Qualitative job demand	1–4	2.62	0.68	0.74	0.26***	0.36***	0.12***	0.18***	0.17***	0.22***	0.23***	0.18***	0.25***
22	Emotional demand	1–4	2.37	0.80	0.87	0.67***	0.66***	0.54***	0.59***	0.52***	0.55***	0.59***	0.44***	0.54***
23	Turnover intention	1–5	2.94	1.10	0.86	0.49***	0.46***	0.52***	0.37***	0.37***	0.37***	0.40***	0.28***	0.46***

		**10**	**11**	**12**	**13**	**14**	**15**	**16**	**17**	**18**	**19**	**20**	**21**	**22**

1	BAT-JC													
2	Exhaustion													
3	Mental distance													
4	Emotional impairment													
5	Cognitive impairment													
6	BAT-JS													
7	Psychological distress													
8	Psychosomatic complaints													
9	MBI-GS													
10	Exhaustion													
11	Cynicism	0.73***												
12	Reduced professional efficacy	0.38***	0.54***											
13	Work engagement	−0.30***	−0.24***	0.27***										
14	Vigor	−0.31***	−0.23***	0.26***	0.93***									
15	Dedication	−0.28***	−0.25***	0.27***	0.95***	0.84***								
16	Absorption	−0.25***	−0.21***	0.24***	0.94***	0.80***	0.84***							
17	Workaholism	0.41***	0.34***	0.29***	0.18***	0.13***	0.17***	0.21***						
18	Working excessively	0.42***	0.34***	0.29***	0.15***	0.09	0.15***	0.17***	0.94***					
19	Working compulsively	0.34***	0.30***	0.25***	0.19***	0.15***	0.17***	0.21***	0.92***	0.73***				
20	Quantitative job demand	0.36***	0.21***	0.15***	0.05	0.01	0.07*	0.06	0.49***	0.54***	0.36***			
21	Qualitative job demand	0.26***	0.17***	0.17***	0.14***	0.09**	0.17***	0.15***	0.37***	0.40***	0.27***	0.65***		
22	Emotional demand	0.62***	0.51***	0.20***	−0.22***	−0.22***	−0.21***	−0.18***	0.35***	0.35***	0.29***	0.36***	0.29***	
23	Turnover intention	0.53***	0.50***	0.10**	−0.41***	−0.39***	−0.38***	−0.38***	0.17***	0.18***	0.13***	0.16***	0.11***	0.45***

SD, standard deviation. ***p < 0.001, **p < 0.01, *p < 0.05.

### Construct Validity

The results regarding the convergent and internal discriminant validity using the MTMM framework are shown in Table 5. Model 1 (CT-CM) had the best fit among the four models, showing a significantly better fit compared with models 2 (NT-CM), 3 (PCT-CM), and 4 (CT-PCM). This hints that the BAT-JC is discriminant and convergent from the MBI-GS. In terms of the values for parameter estimates, all items loaded significantly on the trait factors except for items 2 and 5 of the MBI-GS cynicism, as well as items 2 and 3 of the BAT-JC mental distance. All items loaded significantly on the measurement factors, except for item 1 on the MBI-GS cynicism subscale and items 4 and 5 on the MBI-GS professional efficacy subscale. The estimated correlation values between trait factors were all significant (|r| = from 0.10 to 0.94), except cynicism/mental distance and cognitive impairment (0.07). In terms of method (measurement), the latent correlation between BAT-JC and the MBI-GS was 0.87.

**TABLE 5:** Model fit indices for the multitrait–multimethod framework for the BAT-JC (*N* = 982).

**Model**		**TLI**	**CFI**	**RMSEA[90% CI]**	**χ^2^**	***df***	**Δχ^2^/*df***	***p***	**Modelcomparison**	**Δχ^2^**	**Δ*df***	***p***
**BAT-C (core symptoms)**												
1	CT-CM model	0.89	0.90	0.07 [0.07–0.08]	3779.54	617.00	6.13	<0.001				
2	NT-CM model	0.66	0.68	0.13 [0.12–0.13]	10863.44	664.00	16.36	<0.001	2 vs. 1	7083.899	47	<0.001
3	PCT-CM mode	0.78	0.81	0.10 [0.09–0.10]	6856.83	627.00	10.94	<0.001	3 vs. 1	3077.290	10	<0.001
4	CT-PCM model	0.85	0.87	0.08 [0.08–0.09]	4809.96	618.00	7.78	<0.001	4 vs. 1	1030.425	1	<0.001

*TLI, Tucker-Lewis index; CFI, comparative fit index; RMSEA, root mean square error of approximation; χ2, chi-square; df, degree of freedom; p, p-value; Δχ2, difference in chi-square; Δdf, difference in degree of freedom. CT-CM, correlated traits-correlated methods; NT-CM, no traits-correlated methods; PCT-CM, perfectly correlated traits-correlated methods; CT-PCM, correlated traits-perfectly correlated methods.*

Table 6 shows the results for the external discriminant validity. The AVE of BAT-JC (0.51) was greater than its squared correlations (*R*^2^) with work engagement (0.10) and workaholism (0.19). The AVE of BAT-JS (0.55) was also greater than its squared correlations (*R*^2^) with work engagement (0.04) and workaholism (0.17). These results indicate that the BAT-J can be discriminated from other well-being constructs.

**TABLE 6:** Average variance explained (AVE) and squared latent correlations (*R*^2^) for work engagement, workaholism, and burnout (BAT) (*N* = 982).

**Item**	**AVE**	***R*^2^**	
		**Work engagement**	**Workaholism**
Work engagement	0.69		
Workaholism	0.43	0.04	
BAT-JC	0.51	0.10	0.19
BAT-JS	0.55	0.04	0.17

**FIGURE 2:**
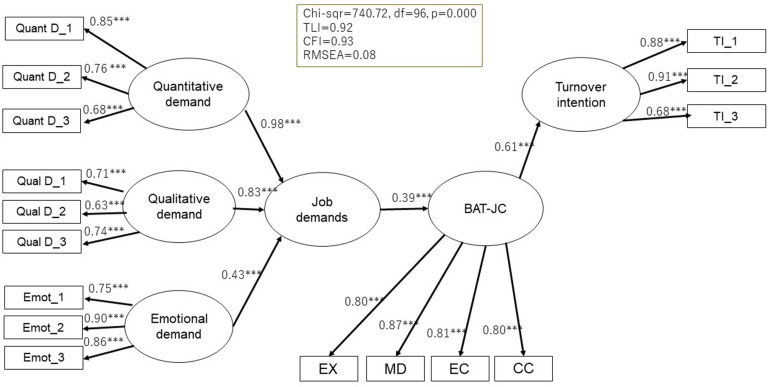
Standardized solution (Maximum Likelihood estimates) of the hypothesized model of the relations of BAT-JC with potential antecedents and consequences (*N* = 982). Quant D, quantitative demand; Qual D, qualitative demand; Emot, emotional demand; TI, Tuniover intention. ***p < 0.001.

**FIGURE 3:**
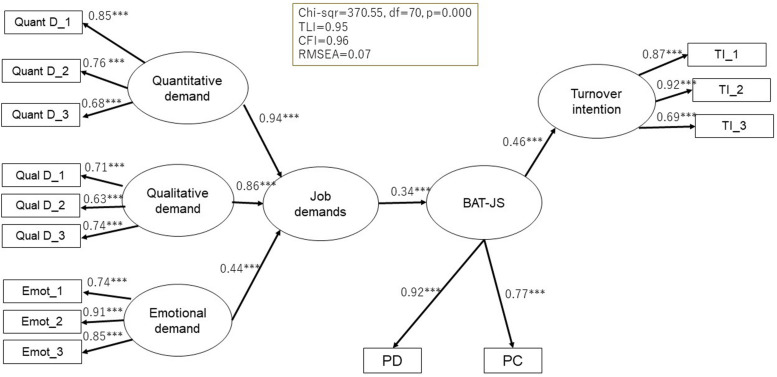
Standardized solution (Maximum Likelihood estimates) of the hypothesized model of the relations of BAT-JS with potential antecedents and consequences (*N* = 982). Quant D, quantitative demand; Qual D, qualitative demand; Emot, emotional demand; TI, Turnover intention. ***p < 0.001.

As for the relations of the BAT-J with potential antecedents and consequences, results of SEM analyses showed that the proposed models (Figures 2, 3) fit adequately with the data. For the BAT-JC, χ^2^(96) = 740.72, TLI = 0.92, CFI = 0.93, and RMSEA = 0.08. For BAT-JS, χ^2^(70) = 370.55, TLI = 0.95, CFI = 0.96, and RMSEA = 0.07. Both the BAT-JC and BAT-JS were positively related to potential antecedents, including quantitative, qualitative, and emotional job demands. Finally, for the relationship with potential consequences, the BAT-JC and BAT-JS were also positively related to turnover intention.

## Discussion

The current study aimed to validate the Japanese version of BAT by evaluating factorial validity, reliability, and construct validity, including convergent and discriminant validity.

For factorial validity, we conducted CFA and ESEM bifactor analysis. For the BAT-JC, BAT-JS, and BAT-J, we compared the goodness of fit of four models, the correlated-factor CFA model, second-order CFA model, bifactor CFA model, and ESEM bifactor model. Results showed that the bifactor ESEM model fits the best to the data. Also, bifactor ESEM analyses showed that the general factor accounted for over two-thirds of the common variance explained for the BAT-JC, BAT-JS, and the BAT-J, indicating a strong general factor. These results suggest that the BAT-JC, BAT-JS, and BAT-J can be treated as a unidimensional scale. This is consistent with the idea that burnout is a syndrome comprising a set of related symptoms referring to one underlying psychological condition, burnout. This also means that each measure may produce a single score for establishing cut-offs to be used as a screening device, which is the added value of BAT-J. Most of the BAT-J items showed significant loadings on the target factors. The exception was when testing the BAT-JS factor loadings, the psychosomatic complaints items displayed strong loadings on the general factor (>0.81) and weak loadings on the specific factor (<0.02). However, when we tested the BAT-J factor loadings, the psychosomatic complaints items showed significant loadings on the target factor (>0.33). These results confirm that the BAT-JC, BAT-JS, and BAT-J can be used as a unidimensional measurement with multidimensional characteristics. Also, all BAT subscales and the composite BAT-JC and BAT-JS showed internal consistency (Cronbach’s α) that exceeded Henson (2001) recommended criterion of 0.70. In fact, the α values of both composite scores and most BAT subscale scores exceeded 0.90.

In terms of test–retest reliability, the stability coefficients of the four BAT-JC subscales, two BAT-JS subscales, and the BAT-JC and BAT-JS composite scores all meet the stringent criterion of 0.50 (Sturman et al., 2005). Therefore, the reliability of BAT-J was confirmed, both in terms of internal consistency as well as test–retest reliability.

For construct validity, we examined the convergent and internal discriminant validity of the BAT-JC vis-ȧ-vis the MBI, using the MTMM framework. In the MTMM model, the latent correlation between the methods (BAT-JC and MBI-GS) was high (0.87), which hints at their convergent validity. This is not surprising because both instruments seek to measure burnout using self-report items scored on a Likert scale. In terms of traits (dimensions), the latent correlations were significant (|r| = from 0.10 to 0.94) except that of cynicism/mental distance and cognitive impairment (0.07); however, the correlations were not perfect, which hints at their discriminant validity. This provides evidence that burnout is a syndrome comprising multiple, interrelated dimensions.

The discriminant validity of the BAT-JC and the MBI-GS is also strengthened by the bivariate correlations (Table 4). The correlation between the BAT-JC and MBI-GS was high (0.73), which is not surprising, as both scales measure burnout. This could be a result of the high correlation between exhaustion subscales (0.85). However, the bivariate correlations between other subscales were low to moderate (ranging from 0.20 to 0.66), especially for reduced professional efficacy, which had a low correlation with other subscales (below 0.29). These results confirmed that, although the BAT-JC is convergent with the MBI-GS, they are not identical, as the constructs they measure differ at the subscale level.

Please note that in the MTMM model, some of the values for parameter estimates and the estimated correlation between traits were negative, suggesting the potential for multicollinearity. Mason and Perreault (1991) pointed out that multicollinearity should not be viewed in isolation, and it is important to consider other factors that influence the accuracy of estimation results and, thus, may either aggravate or mitigate the deleterious effects of multicollinearity. Also, if the measure were highly reliable, the harmful effects of multicollinearity could be largely offset (Grewal et al., 2004). The current study sample size was large enough (*n* = 982), and the Cronbach’s α of the subscales and the compounded BAT-JC scale exceeded 0.86; thus, our measure was reliable. We could, therefore, conclude that multicollinearity was offset.

As for external discriminant validity, the AVEs of the BAT-JC and BAT-JS were greater than their respective squared correlations (*R*^2^) with work engagement and workaholism. This result confirms that the BAT-J assesses a different construct than work engagement and workaholism, confirming the external discriminant validity of the BAT-J.

For further construct validity, the BAT-JC and BAT-JS showed positive relationships with several possible antecedents and consequences. These results were in line with the JD-R model, which assumes that high job demands are associated with high levels of stress, health problems, and poor organizational outcomes (Demerouti et al., 2001). Thus, the construct validity of the BAT-J was confirmed.

These results provide evidence that burnout comprises multidimensional, inter-correlated dimensions that cannot be grasped by the MBI, indicating that the BAT-J provides a more detailed understanding of burnout characteristics and can be an alternative measure for assessing burnout.

### Limitations and Future Directions

The current study has three limitations that warrant future research. First, sampling bias might exist, as we included only healthy employees in our study. As the BAT was also developed for assessing severe burnout, we need to confirm its validity and reliability in a sample of employees with burnout as well.

Second, it is necessary to establish appropriate cut-offs for screening employees who are at risk of burnout. In the original study in Belgium, cut-offs had already been established (Schaufeli et al., 2019, Manual BAT. KU Leuven, Belgium: unpublished internal report 78). Because levels of burnout vary across cultures and nations (Savicki, 2002), nation-specific cut-offs should be developed (Schaufeli and Van Dierendonck, 1995), also for Japan. This study’s findings confirmed that the BAT-JC, BAT-JS, and BAT-J could produce a single score; the next step is to develop a Japan-specific cut-off to use the BAT as a screening device.

Third, we need to examine the usability of a BAT-JC and BAT-JS combination in research and practice. As explained previously, the BAT-JC represents the core burnout symptoms, and the BAT-JS represents the secondary burnout symptoms. To what extent the BAT-JS improves the assessment of burnout over and beyond the BAT-JC is an open question. Also, future research should explore whether secondary symptoms always appear simultaneously with core symptoms or only when core symptoms reach a certain level of severity.

## Conclusion

The results of the current study provide primary evidence for the factorial validity, reliability, and construct validity of the BAT-J. This tool was developed to overcome various flaws in the MBI-GS related to the conceptualization and dimensionality of burnout, as well as its practical applicability. For practical use, this study confirmed that the BAT-J can be used as a unidimensional measurement and can produce a single score for establishing a cut-off to be used as a screening device in the next step. The BAT-J may be a viable alternative to the MBI-GS in research and practice in Japan.

## Data Availability Statement

The datasets generated for this study are available upon request to the corresponding author.

## Ethics Statement

The Ethics Review Board of Toyo University approved the procedures before starting the study. Participants had the option of not responding to any part of the questionnaire at any time and to discontinue the survey at any point. Participants’ consent was confirmed based on their completion of the questionnaire.

## Author Contributions

KS was responsible for the data analysis and writing the draft of the manuscript. AS planned the research design as a principal investigator of the project and contributed to the writing of the manuscript. HT was responsible for the data collection, data analysis for CFA and ESEM bifactor analysis, and contributed to the writing of the manuscript. WS was involved in the original conceptualization of the work and reviewed various versions of the manuscript. All authors contributed to the article and approved the submitted version.

## Conflict of Interest

The authors declare that the research was conducted in the absence of any commercial or financial relationships that could be construed as a potential conflict of interest.

## Footnotes

1 For more details on the development of the new burnout instrument (see Schaufeli et al., 2019).

## Abbreviations

AIC: Akaike Information Criterion
AVE: average variance explained
BAT: Burnout Assessment Tool
BAT-C: Burnout Assessment Tool – core symptoms
BAT-J: Japanese version of the Burnout Assessment Tool
BAT-JC: Japanese version of the Burnout Assessment Tool – core symptoms
BAT-JS: Japanese version of the Burnout Assessment Tool – secondary symptoms
BAT-S: Burnout Assessment Tool – secondary symptoms
BIC: the Bayesian Information Criterion
CFA: confirmatory factor analysis
CFI: comparative fit index
CT-CM: correlated traits-correlated methods
CT-PCM: correlated traits-perfectly correlated methods
EFA: exploratory factor analysis
ESEM: exploratory structural equation modeling
MBI: Maslach Burnout Inventory
MBI-GS: Maslach Burnout Inventory – General Survey
MTMM: multitrait–multimethod
NT-CM: no traits-correlated methods
PCT-CM: perfectly correlated traits-correlated methods
RMSEA: root mean square error of approximation
TLI: Tucker–Lewis Index.
