# Supplementary material for: Validation of the Japanese Version of the Burnout Assessment Tool
Source: Front Psychol. 2020 Aug 11;11:1819. doi: 10.3389/fpsyg.2020.01819 (PMC7431961; doi:10.3389/fpsyg.2020.01819)
Supplement: Supplementary file 1 [file Data_Sheet_1.pdf]

Appendix

Burnout Assessment Tool

Instruction

The following statements are related to your work situation and how you experience this situation. Please state how often each statement applied to you.

Scoring

1. Never 2. Rarely 3. Sometimes 4. Often 5. Always

Items

Core symptoms

Exhaustion
At work, I feel mentally exhausted
Everything I do at work requires a great deal of effort
After a day at work, I find it hard to recover my energy
At work, I feel physically exhausted
When I get up in the morning, I lack the energy to start a new day at work
I want to be active at work, but somehow I am unable to manage
When I exert myself at work, I quickly get tired
At the end of my working day, I feel mentally exhausted and drained
Mental distance
I struggle to find any enthusiasm for my work
At work, I do not think much about what I am doing and I function on autopilot
I feel a strong aversion towards my job
I feel indifferent about my job
I’m cynical about what my work means to others
Cognitive impairment
At work, I have trouble staying focused
At work I struggle to think clearly
I’m forgetful and distracted at work
When I’m working, I have trouble concentrating
I make mistakes in my work because I have my mind on other things

Emotional impairment
At work, I feel unable to control my emotions
I do not recognize myself in the way I react emotionally at work
During my work I become irritable when things don’t go my way
I get upset or sad at work without knowing why
At work I may overreact unintentionally

Secondary symptoms

Psychological complaints
I have trouble falling or staying asleep
I tend to worry
I feel tense and stressed
I feel anxious and/or suffer from panic attacks
Noise and crowds disturb me
Psychosomatic complaints
I suffer from palpitations or chest pain
I suffer from stomach and/or intestinal complaints
I suffer from headaches
I suffer from muscle pain, for example in the neck, shoulder or back
I often get sick
